# Tumor Microenvironment-Stimuli Responsive Nanoparticles for Anticancer Therapy

**DOI:** 10.3389/fmolb.2020.610533

**Published:** 2020-12-18

**Authors:** Reju George Thomas, Suchithra Poilil Surendran, Yong Yeon Jeong

**Affiliations:** ^1^Department of Radiology, Chonnam National University Hwasun Hospital, Hwasun, South Korea; ^2^BioMolecular Theranostics (BiT) Laboratory, Department of Biomedical Sciences, Chonnam National University Medical School, Chonnam National University Hwasun Hospital, Gwangju, South Korea

**Keywords:** stimuli, nanoparticle, tumor microenvironment, cancer, drug release

## Abstract

Cancer is a disease that affects a large number of people all over the world. For treating cancer, nano-drug delivery system has been introduced recently with objective of increasing therapeutic efficiency of chemotherapeutic drug. The main characteristics of this system are the encapsulation of the insoluble chemotherapeutic cargo, increasing the period of circulation in the body, as well as the delivery of the drug at that specific site. Currently, the nano-drug delivery system based on the stimuli response is becoming more popular because of the extra features for controlling the drug release based on the internal atmosphere of cancer. This review provides a summary of different types of internal (pH, redox, enzyme, ROS, hypoxia) stimuli-responsive nanoparticle drug delivery systems as well as perspective for upcoming times.

## Introduction

Cancer is a common cause of death in humans. To overcome cancer, the general strategies used in chemotherapy still have some specific limits, such as adverse side effects because of unintentional drug accumulation in normal cells and not cancer cells. The discovery of nanomedicine, which is quite impactful for treating cancer, has created a new stage for targeted therapeutics. The featured mechanism of nanoparticles for targeting the tumor region is the enhanced permeation and retention (EPR) effect (Cheng et al., 2014; Shi et al., 2017). The capability of nanoparticles to accumulate at the tumor site through the effect of EPR is determined by the shape, size, and surface chemistry of the nanoparticles (Blanco et al., 2015). Additionally, some other aspects lead to the uptake of nanoparticles by tumor cells as well as consequent drug release at the tumor site. Stimuli-responsive nanoparticulate systems can deliver molecules of therapeutic drugs without affecting the regions near the tumor site (Ruttala et al., 2018). These stimuli-responsive particles used in chemotherapy for treating cancer have become very popular, along with the evolution of nanotechnology, nanomedicine, and material chemistry (Mura et al., 2013; Torchilin, 2014).

**Figure 1:**
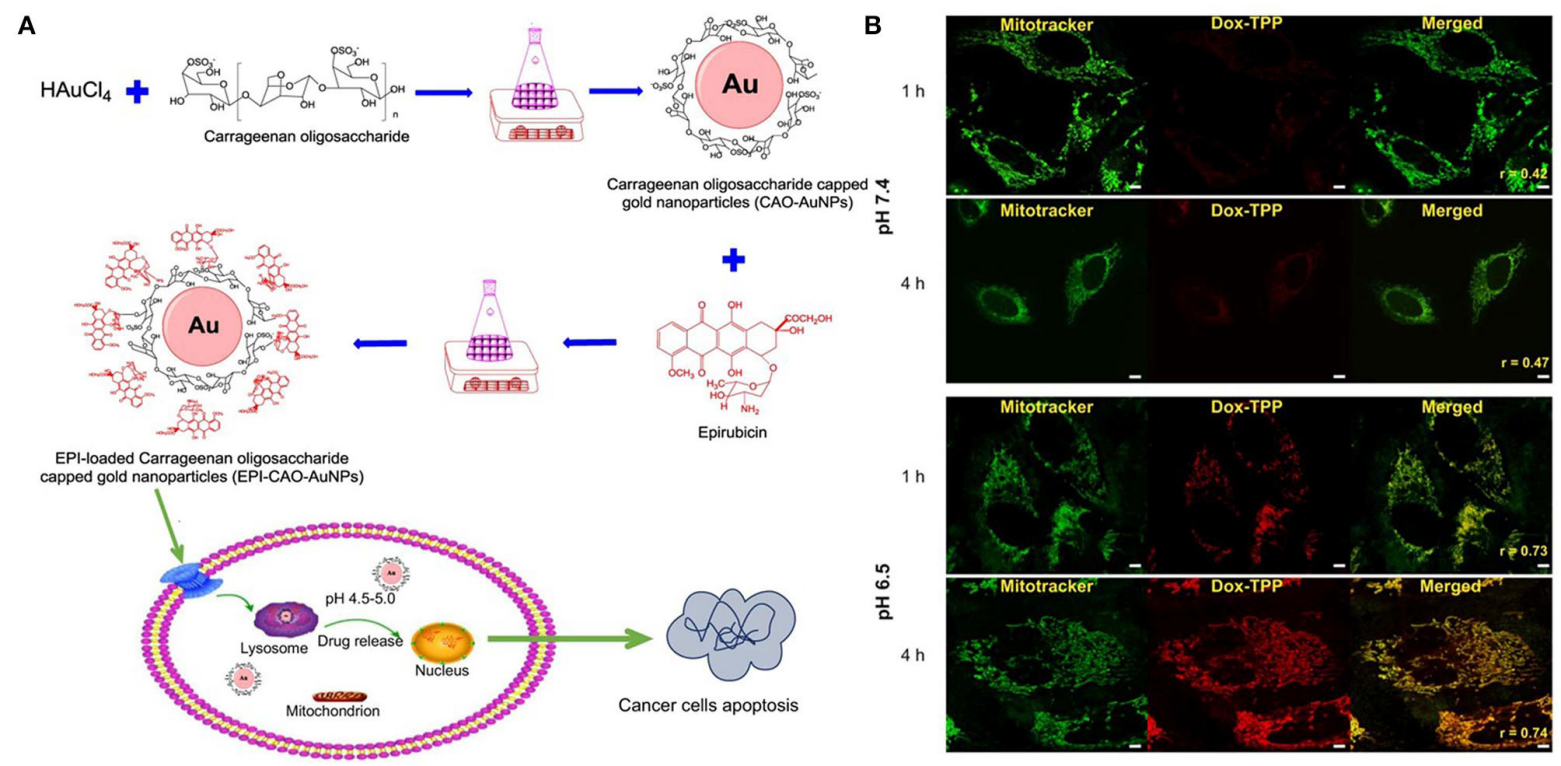
pH stimuli responsive system of **(A)** epirubicin loaded Carrageenan oligosaccharide capped gold nanoparticle. **(B)** Drug release from the Dox-TPP nanoparticle was observed at lower pH (6.5) in the intracellular compartment of cell whereas at 7.5 pH minimal release was observed after 4 h. Reprinted with permission from Chen X. et al. (2019) and Palanikumar et al. (2020). Copyright © 2017 OMICS International.

**Scheme 1:**
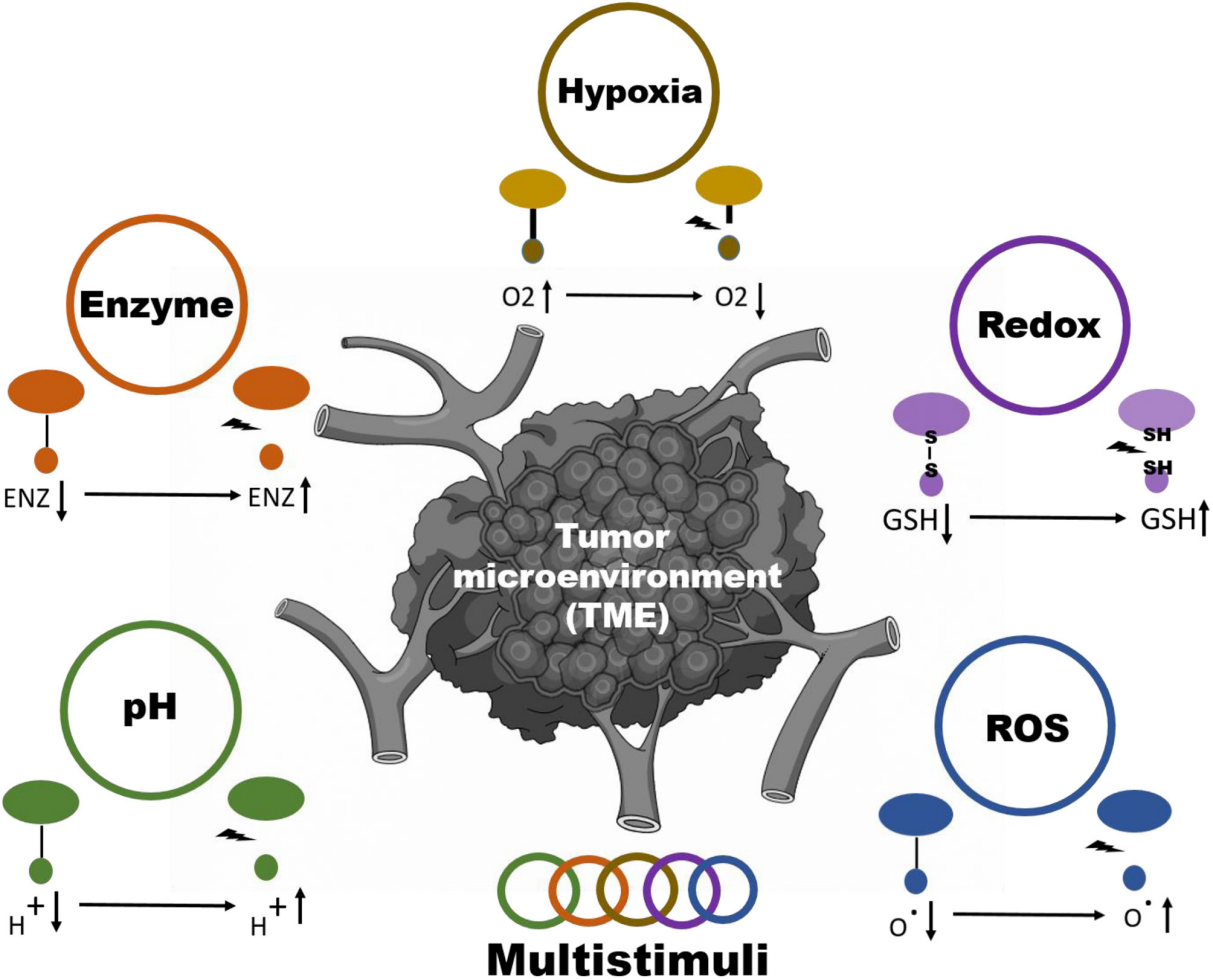
Schematic illustration for internal stimuli-responsive drug delivery systems.

Stimuli responsive nanoparticles systems are divided into 2 classes: internal (pH, enzyme, ROS, hypoxia, redox) and external (radiation, electromagnetic, thermal) stimuli depending upon the method of inducing the delivery of the drug (Figure 1) (Taghizadeh et al., 2015; Yao et al., 2016). A vital role is also played by the design, material, and nanoparticle chemistry for spatiotemporally controllable delivery of the drug in addition to some of the stimuli-inducing factors (Crucho, 2015; Du et al., 2015; Ding et al., 2016; Wang S. et al., 2016). Rapid and controllable release of the drug through tuning the features of nanoparticles are the two main benefits of stimuli-responsive nanoparticle systems (Liao et al., 2015). Although there are few disadvantages of external stimuli-responsive materials, they are limited in terms of their tumor targeting capability to induce the proper release of the drug. An internal stimulus at the site of the tumor could address this issue. In this review, we summarize the current strategies to achieve tumor microenvironment (TME)-responsive nanoparticle drug release (Scheme 1). We also cover the nanoparticle properties required to respond to internal stimuli, such as pH, enzyme, ROS, hypoxia, and redox conditions, in detail.

## Internal Stimuli-Responsive System

### pH-Responsive System

Compared to the other methods of drug release, the release of the drug from the nanoparticles in response to the pH can be considered the most efficient method. This particular mechanism for releasing drugs based on the slightly acidic microenvironment of the tumor as well as extremely acidic intracellular compartments with a pH value of 5.5 is known as pH-responsive release mechanism (Yu J. et al., 2014). The microenvironment of a tumor is acidic because of the built-up lactic acid formed during the division of the tumor cells. This specific phenomenon is called the Warburg effect (Vander Heiden et al., 2009; Liberti and Locasale, 2016). Nanoparticles with pH sensitivity in this acidic environment trigger a stimuli-responsive action, which consequently changes the chemistry of the material and induces the drug release (Gao et al., 2010) (Table 1).

**Table 1:** pH stimuli responsive nanoparticle for anti-cancer drug release.

**Nanoparticular components**	**Mechanism**	**Payload**	**References**
Phospholipid, polyurethane	Acetal link destabilization	Doxorubicin	Deng et al., 2014
Polyethelene glycol, polycaprolactone	β-carboxylic amides link hydrolyzation	Doxorubicin	Gao et al., 2014
Poly-L-Leucine, poly-L-Lysine	β-carboxylic amides link hydrolyzation	Doxorubicin	Han et al., 2015
Poly-l-Arginine, polydopamine	bis-norbornene as acid-labile linker destabilization	Doxorubicin	Yu Y. et al., 2014
Polyethelene glycol, hyaluronic acid	Calcium phosphate dissolution	Doxorubicin	Han et al., 2013
Hyaluronic acid	pH stimuli sensitive lipids	Doxorubicin	Wang Z. et al., 2016
Zirconia	Molecular switching	Doxorubicin	Wang M. et al., 2016
Gold	pH stimuli responsive coating	Epirubucin	Chen X. et al., 2019
Polylactic glycolic acid	Acidity triggered rational membrance destabilization	Doxorubicin	Palanikumar et al., 2020

To produce pH-responsive nanoparticles, both organic and inorganic materials are used (Gisbert-Garzaran et al., 2017). The pH-sensitive systems are prepared with polymer-based nanoparticles because their pH-induced changes, such as changes in volume or conformation and solubility, can be easily obtained. Polymer systems with pH-based linkers will experience a change in their properties based on charge reversal when the pK value of the polymer changes. Cationic polymers change from being hydrophobic to hydrophilic, whereas anionic polymers change from hydrophilic to hydrophobic. Examples of pH-responsive cationic polymers are poly(histidine), poly(4-vinyl pyridine), poly(β-amino ester), and poly[2-(diisopropylamino) ethyl methacrylate]. Similarly, anionic polymers change to hydrophobic from hydrophilic when exposed to a lower pH. Examples of anionic polymers are sulfonamide-based polymers, poly(aspartic acid) (PAsp), and poly(methacrylic acid) (PMAA). Another strategy of achieving a pH-induced release mechanism is the inclusion of linkers which hydrolyze at low pH and therefore become unstable. The linkers commonly used in cancer drug delivery systems are β-thiopropionate moieties, orthoesters, cis-aconityl groups, acetal/ketal groups, imine, and hydrazine (Deirram et al., 2019).

Doxorubicin-loaded phospholipid-linked polyurethane nanoparticles were prepared with acetal linkers, which were responsible for the particle degradation in acidic conditions. The molecular dynamics of these micelles revealed a compact core with surface-attached polymer chains and encapsulated doxorubicin (John et al., 2016). Doxorubicin-loaded polyethylene glycol (PEG) methyl ether-block-poly(ε-caprolactone) nanomicelles were prepared using different β-carboxylic amide amounts to improve the acid-labile properties of the polyester moieties (Deng et al., 2014). β-carboxylic amides were exposed at a low pH of 6.0 to induce their hydrolysis, leading to a charge reversal from negative to positive of the polymeric micelles. Another benefit of the charge reversal is the increased uptake by the tumor cells, which has been previously shown with the HepG2 liver cancer cell line. The same kind of charge reversal method was employed in another study with β-carboxylic amide. Tat peptide was amidated to produce succinyl chloride [Tat (SA)] by click chemistry. PLLeu–PLL (DMA)–Tat (SA) nanomicelles were prepared with the nuclei-targeting Tat peptide (Han et al., 2015).

Bis-norbornene, as an acid-labile linker in the ring-opening metathesis polymerization, is used for preparing a polymer-drug conjugate of doxorubicin (DOX) and PEG (Gao et al., 2014; Yu Y. et al., 2014). Another method for releasing the drug from a nanoparticle used self-assembled hyaluronic acid (HA) mineralized by calcium phosphate, resulting in the formation of hydroxyapatite nanoparticles loaded with DOX. When the pH was low, the mineral dissolved; thus, the drug was released into the microenvironment of the tumor (Han et al., 2013). HA pH-responsive lipid membrane mesoporous silica nanoparticles (MSNs) have been utilized as a CD44^+^-based targeting system for cancer therapy. DOX was loaded into these pH-sensitive MSNs, which were unstable in acidic conditions, thus releasing DOX from the MSN cage. This particular system is considered very biocompatible due to the use of silica, biocompatible lipids, and HA (Wang Z. et al., 2016). A dual pH-responsive zirconia ceramic nanosystem was prepared. The dual pH-mediated sensitivity was attributed to the hollow mesoporous zirconia nanospheres releasing the DOX payload through a molecular switching function (Wang M. et al., 2016).

In another system based on gold nanoparticles, carrageenan oligosaccharide (CAO) was used as a reducing agent to synthesize CAO-coated gold nanoparticles (AuNPs) (Figure 1). Epirubicin (EPI) was loaded into the nanoparticles as an anticancer drug. The *in vitro* release of EPI induced the apoptosis of HCT-116 and HepG2 cells (Chen X. et al., 2019). DOX-triphenylphosphonium (DOX-TPP) was loaded inside BSA-PLGA nanoparticles, and ATRAM was conjugated to induce tumor targeting capabilities. The pH-responsive effect was attributed to the ester bond hydrolysis in PLGA, which resulted in drug release (Palanikumar et al., 2020).

In the low-pH environment of tumors, a supersensitive polymer material, poly(2-ethyl-2-oxazoline)-poly(methacryloyl sulfadimethoxine), was synthesized (PEOz-*b*-PSD). PEOz-*b*-PSD and polyamidoamine/DOX can form nanoparticles at the physiological environment. This nanoparticles were protonated and underwent a charge reversal resulting in the detachment of PEOz-b-PSD, which formed ultrafine nanoparticles with improved accumulation in tumors. The ultrasensitive nanoparticles in the tumor microenvironment showed to be beneficial for enhancing the treatment efficacy of DOX in solid tumors (Jia et al., 2020). An innovative nanorobot was developed based on iron oxide nanoparticles (Fe_3_O_4_) chemically conjugated to carbon nanotube loaded with doxorubicin and anti-epithelial cell adhesion molecule antibody for targeting to colorectal cancer. This nanoparticle system provided mechanism whereby Fe_3_O_4_ moeity opening facilitated doxorubicin release from carbon nanotube. The therapeutic effect wqs tested in colon cancer spheroids (HCT116) and found to have sufficient anti-cancer effect (Andhari et al., 2020).

### Redox-Responsive Systems

Compared to normal cells in our body, cancer cells have a higher redox potential with nearly 100–1,000-fold upsurge. Glutathione (GSH) is responsible for the destabilization of the redox-mediated disulfide linkage. The variation in the level of GSH in the blood as well as the environment of the tumor is nearly 500-fold, which can be utilized for efficient internal stimuli-responsive drug release. Redox-responsive nanoparticles have a disulfide bond-linked removable shell, which can easily be shed in the presence of the GSH enzyme, resulting in the release of the drug (Bauhuber et al., 2009; Jhaveri and Torchilin, 2014). Two main types of redox-responsive bonds are utilized in redox-responsive delivery systems: delivery systems with disulfide bonds and diselenium bonds. These disulfide bonds are cleaved to form sulfhydryl groups from glutathione, which indirectly results in the breakdown of the polymer system containing these bonds, thereby releasing the drug. In a similar manner, diselenium bonds can be cleaved more efficiently than disulfide bonds because their bond energy is lower than that of disulfide bonds (Gunawan et al., 2014; Guo et al., 2018) (Table 2).

**Table 2:** Redox stimuli responsive nanoparticle for anti-cancer drug release.

**Nanoparticular components**	**Mechanism**	**Payload**	**References**
Poly(3-caprolactone), poly(N,N-dimethylamino-2-ethylmethacrylate)	Disulphide link destabilization	Doxorubicin	Qu et al., 2017
Au, mesoporous silica	Host-guest interaction system release of the hydrophilic Fc+	Doxorubicin	Li et al., 2015
Mesoporous silica	Disulphide link destabilization	Doxorubicin	Xiao et al., 2015
Mesoporous silica	Disulphide link destabilization	Doxorubicin	Li et al., 2020
Polyethylene glycol	Ditelluride link destabilization	Doxorubicin	Pang et al., 2020
Polydopamine	Disulphide link destabilization	Doxorubicin	Tian and Lei, 2019

A redox-sensitive PEGylated liposome with orthoester linkages was prepared. The poly-e-benzyloxycarbonyl-L-lysine (PzLL) as the lipid portion is responsible for the formation of micelles of DOX-loaded mPEG-SS-PzLL nanomicelles. A special property of this nanomicelle is its ability to shed its PEG shell at an increased level of GSH, which is accompanied with DOX release (Wen et al., 2011). This particular strategy for detaching the shell was utilized in disulfide-linked poly(3-caprolactone)-b-poly(N,N-dimethylamino-2-ethylmethacrylate), which showed a GSH-dependent release of DOX along with the gene. These positively charged nanoparticles could bind DNA and could be taken up by cells (Li et al., 2014).

MSNs incorporate redox responsiveness with AuNP-like gatekeepers (Qu et al., 2017). Here, rather than the conventional GSH reactive disulfide linkage, a host-guest interaction system was used, which involved cyclodextrin-modified AuNPs bound to the MSN for drug release. The role of the host was played by the molecules of ferrocene (Fc), which were bound to the cyclodextrin (guest) and responded to the redox conditions by releasing hydrophilic Fc+, thus opening the gate for the subsequent release of the drug from the cavities. Another MSN system used Arg-Gly-Asp (RGD) peptide as a capping agent. RGD as the capping agent functioned as a targeting moiety in redox-sensitive peptide-functionalized MSNs (DOX@MSN-S-S-RGD). This system showed improved cellular uptake in the COS-7 cells as well as improved DOX release (Li et al., 2015).

A redox-responsive MSN was developed as a drug nanocarrier by non-covalent functionalization of MSNs with amphiphilic peptides containing the RGD ligand. After the internalization of MSNs by cancer cells *via* the receptor-mediated endocytosis, the surface amphiphilic peptides and alkyl chain of redox responsive MSN/DOX were removed to induce rapid drug release intracellularly after the cleavage of the disulfide bond triggered by GSH (Xiao et al., 2015). The protein capping and the drug release strategy were also studied for “transferrin-capped MSNs (DOX@MSNs-S-S-Tf), which showed cancer-targeting effects and released DOX through uncapping transferrin because of disulfide link destabilization. This specific system claims the slower release of the drug without GSH and rapid release in an intracellular state (Li et al., 2020). DOX-releasing nanoparticles were developed with MSNs coated with disulfide linkers to regulate the drug release in response to varying redox conditions.

**Figure 2:**
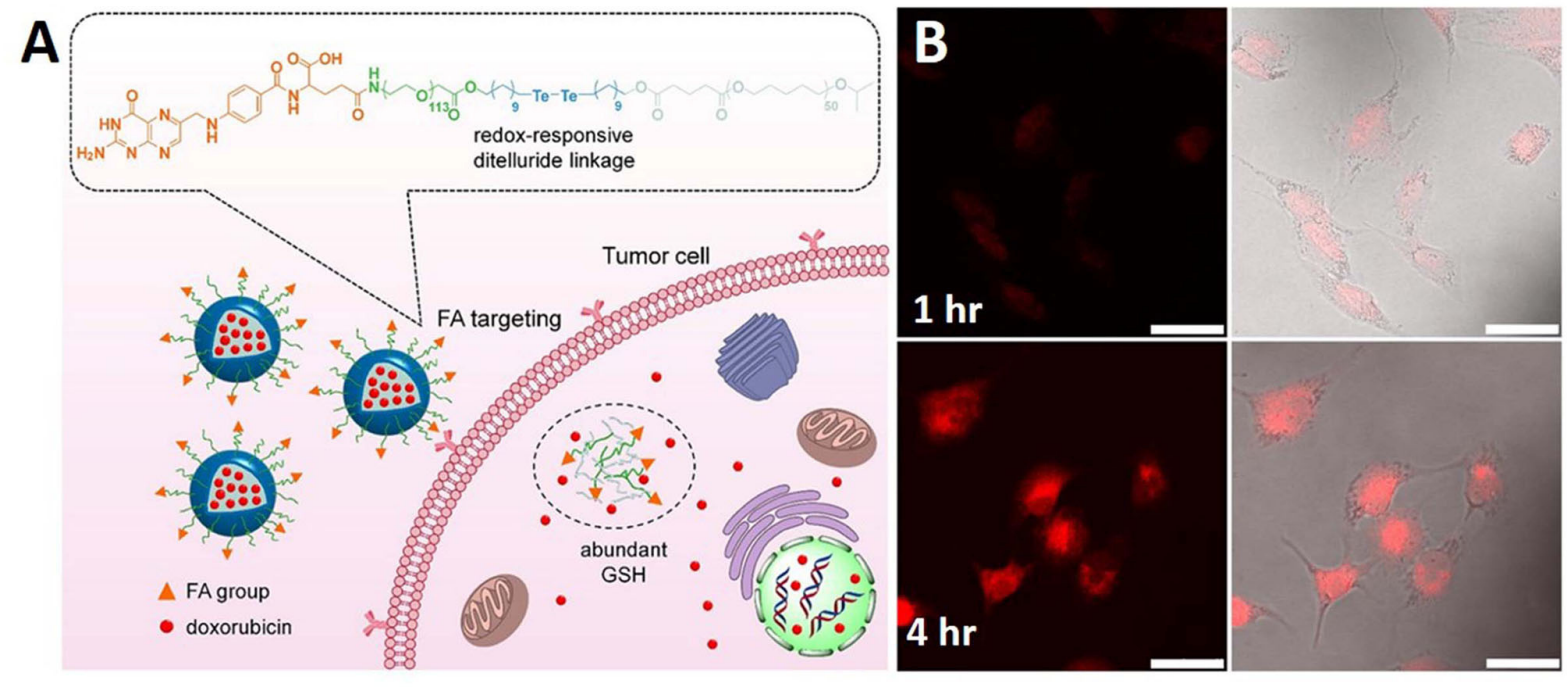
Schematic illustration of **(A)** redox sensitive NPs with disulfide linked PEG shell which can respond to tumor intracellular GSH microenvironments for controlled release of therapeutic agents. **(B)** PDA@PMAA nanoparticles kept for 1 h and 4 h time period. Reprinted with permission from Pang et al. (2020) and Tian and Lei (2019). Copyright © 2020 Frontiers Media S.A.

In another study, GSH-responsive ditelluride bonds were utilized to synthesize nanoparticles releasing loaded doxorubicin in cancer cells. Compared to the S-S bond, the detelluride bond has a lower bond energy. PEGylated folic acid (FA) and redox responsive ditelluride linkage created an ambiphilic system capable of loading doxorubicin (F-TeNP_DOX_). An *in vivo* tumor treatment study showed significant tumor reduction in the F-TeNPDox nanoparticle-injected group compared to the other groups. A redox labile coating of disulfide-crosslinked poly(methacrylic acid) (PMAA) was coated onto polydopamine (PDA) nanoparticles prepared by the precipitation polymerization method. In addition to redox-responsiveness, PDA induced responsiveness to photothermal stimuli. Through the dual stimuli-responsive mechanism of the PDA@PMAA nanoparticles, 86% DOX release was achieved. This nanoparticles were found to be highly toxic in response to both stimuli (redox and photothermal) (Figure 2) (Pang et al., 2020). Nanoparticles based on poly(methacrylic acid) with disulfide crosslinkers were synthesized to load drugs and release them in response to redox stimuli. The nanoparticle core consisted of PDA, which has a very high photothermal efficiency. This nanoparticle simultaneously acted as a chemotherapeutic and photothermal agent (Tian and Lei, 2019).

### Enzyme-Responsive Systems

An ailing condition, such as cancer, can lead to changed enzyme expression, which can cause higher levels of matrix metalloproteinases (MMPs), cathepsin, hyaluronidase, trypsin, thrombin, proteinase K, etc. The upregulated enzymes can be utilized for the controlled release of cargos from nanocarriers, breakage of polymer backbones, physical disruption of the nanocarriers and cleavage of bonds between the drug and the carrier in the tumor microenvironment (Mi, 2020). The biorecognition and catalytic function of enzymes can be used for the destabilization of nanoparticles. The major classifications of enzymes include hydrolase (protease, trypsin, elastase, lipase, glycosidase) and oxidoreductase (glucose oxidase, peroxidase, azoreductase, glutathione reductase) (Table 3). Hydrolase enzymes are capable of cleaving chemical bonds by the addition of water molecules; oxidoreductase enzymes are capable of catalyzing oxidation/reduction reactions, resulting in the destruction of the nanoparticles (Rabiee, 2019).

**Table 3:** Enzyme stimuli responsive nanoparticle for anti-cancer drug release.

**Nanoparticlular components**	**Mechanism**	**Payload**	**References**
Polyethylene glycol	Enzyme responsive linker glycylphenyl-lananylleucy glycine tetrapeptide	Paclitaxel	Li et al., 2017
L-lysine polyurethanes	Nanoparticle degradation by lysosomal esterase enzyme	Doxorubicin	Joshi et al., 2019
Poly(ester-urethane)	Enzyme responsive amphiphilic poly(ester-urethanes)	Camptothecin	Joshi et al., 2019
Mesoporous silica	Chitosan with an azo bond destabilization	Doxorubicin	Cai et al., 2020
Hyaluronic acid	HAase enzyme mediated destabilization of nanoparticle	Doxorubicin	Naz et al., 2019

Paclitaxel (PTX) was delivered using Janus PEGylated peptide dendrimer nanoparticles for the treatment of breast cancer. The tumor microenvironment enzyme-responsive linker glycyl phenylalanine leucyl glycine tetrapeptide was incorporated into the nanoparticles for effective release of PTX. The nanoparticles were destabilized in the presence of enzyme capthepsin B overexpressed in breast cancer, which acts as a lysosomal cysteine protease. The cell uptake and PTX release study also indicated the effectiveness of the nanoparticle in delivering PTX and the release of the drug into the tumor microenvironment using an enzyme degradable linker (Li et al., 2017).

Polyurethanes have been used as an enzyme stimuli-responsive carrier system for delivering cargos to the tumor microenvironment. In the related study, L-lysine-based amphiphilic enzymes and thermosensitive nanocarriers were designed, which were capable of delivering the drug DOX to the intracellular region of the cancer tissue at physiological temperature. The cumulative DOX release was studied in the presence and absence of intracellular esterase enzyme, which showed that a considerable amount of the drug was released in the presence of the enzyme. All the *in vitro* studies showed effective drug release in the presence of enzyme at a temperature of 42°C, which indicated the dual responsive characteristics of the nanoparticles. Overall, the utilization of L-lysine polyurethanes as a dual sensitive drug delivery system for delivering anticancer drugs could be a useful tool for cancer treatment (Joshi et al., 2019). Another study used polyurethanes containing DOX and camptothecin (CPT). In this study, enzyme-responsive amphiphilic poly(ester-urethanes) were synthesized by polycondensation of L-tyrosine amino acid moieties and hydrophilic PEG. Enzyme-dependent cumulative release of both DOX and CPT was observed when biological amounts of esterase enzyme were present. The amphiphilic polyurethanes showed no toxicity toward the normal cell lines, but they were toxic to the cancer cell lines when treated with the anticancer drugs DOX and CPT (Aluri and Jayakannan, 2017).

**Figure 3:**
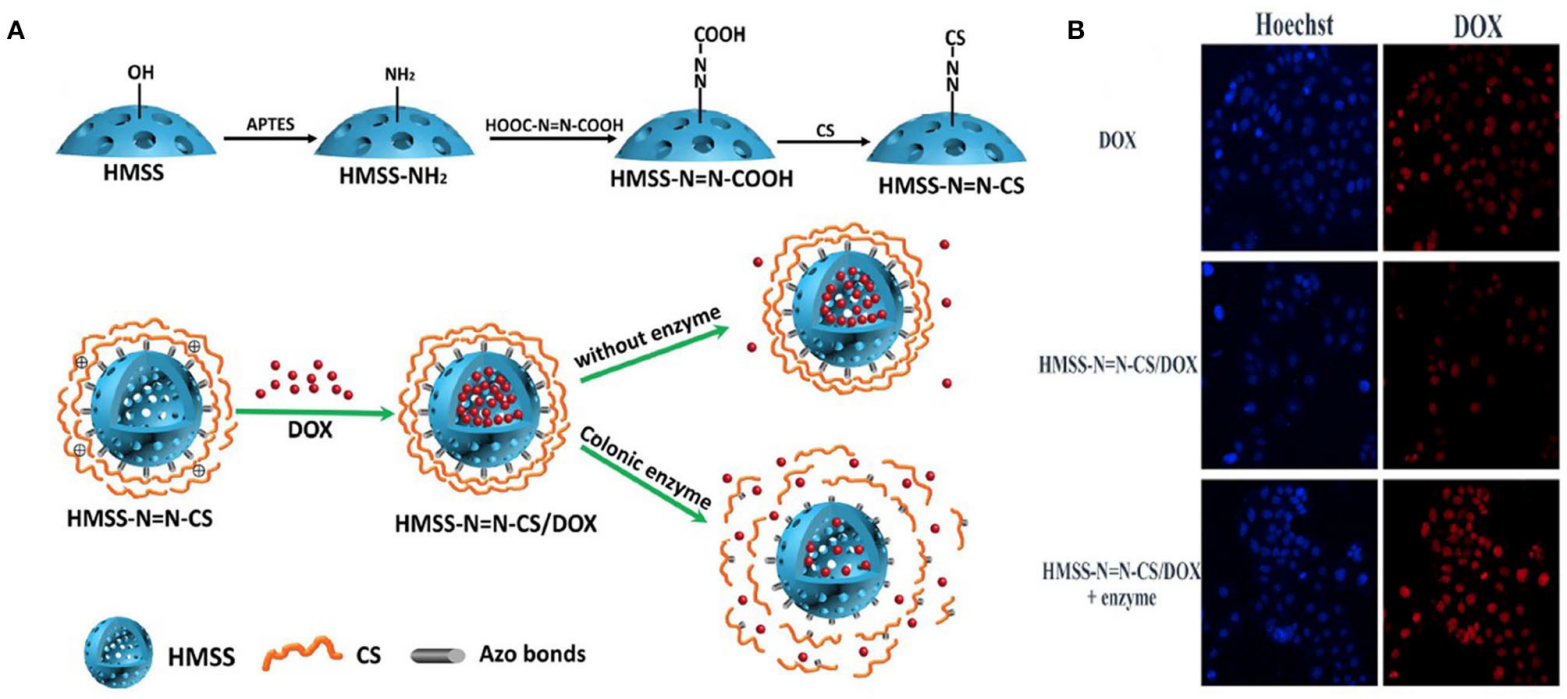
Schematic illustration of preparation of HMSSN=N-CS. **(A)** Drug loading and colon enzyme responsive release of DOX from HMSS-N=N-CS, CLMS of Caco-2 cells incubated with HMSS/DOX in presence and absence of enzyme compared with DOX only. **(B)** MFI of DOX, HMSS/DOX, and HMSS/DOX +Enzyme using FCM analysis. Reprinted with permission from Cai et al. (2020). This article is licensed under a Creative Commons Attribution 4.0 International License.

Hollow mesoporous silica spheres (HMSS) for drug delivery have been studied widely. In one study, HMSS was modified using chitosan with an azo bond responsive to the enzyme activity in the TME. DOX was encapsulated in the hollow cavity, which was released in the presence of colon enzyme. The drug release profile of the HMSS/DOX was investigated, and a sustained release of the drug in the presence of the enzyme was found when compared to the release in the absence of enzyme. The *in vitro* results supported the drug release studies, which showed the ability of the nanoparticles to release the drug at the TME (Figure 3) (Cai et al., 2020).

**Figure 4:**
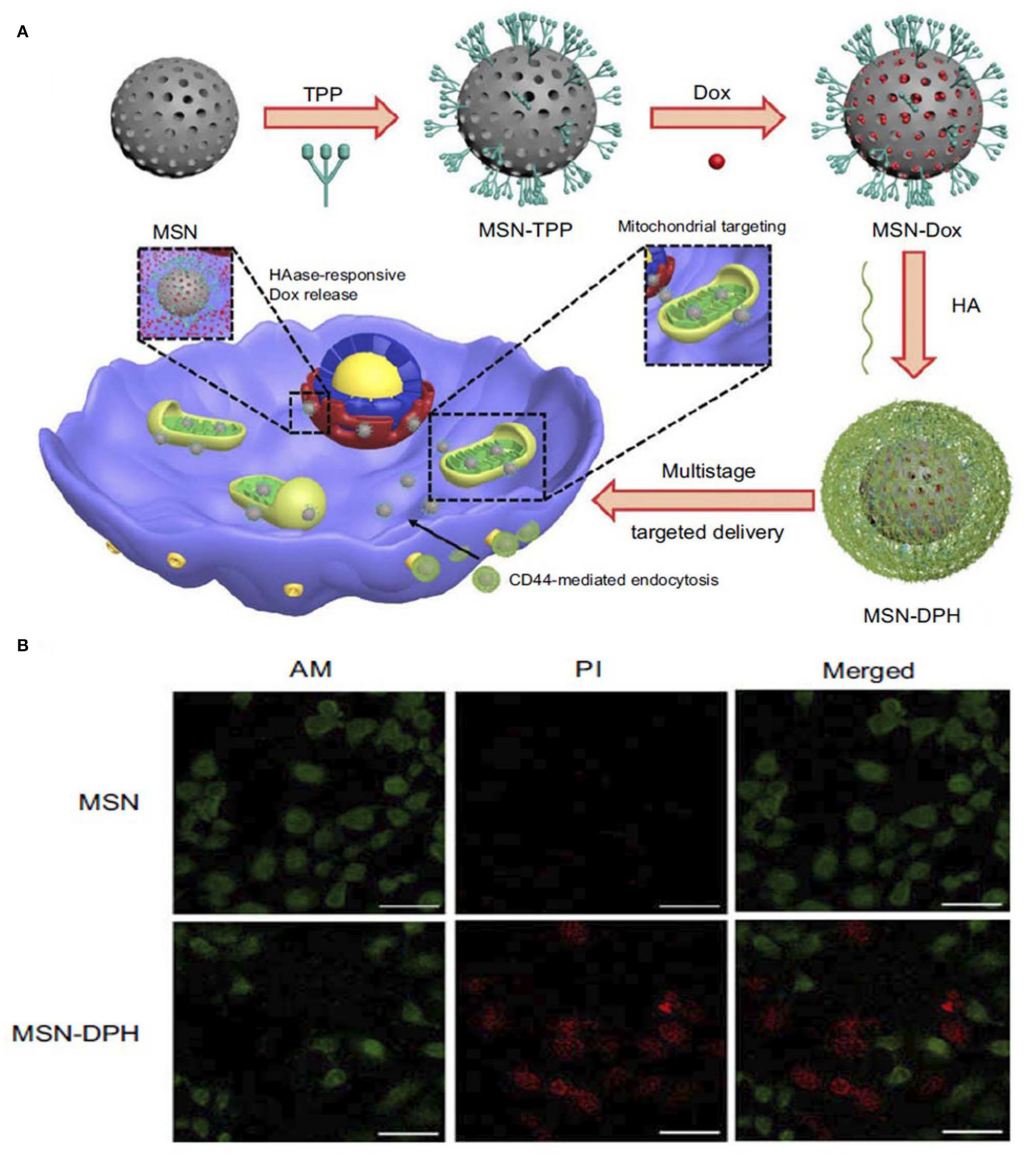
Schematic illustration of synthesis and application of **(A)** MSN-DPH nanoparticles, Cumulative DOX release from MSN-DOX, MSN-DPH, and MSN-DPH+HAase. **(B)** Live/dead images of MGC-803 cells incubated with MSN and MSN-DPH nanoparticles. Reprinted with permission from Naz et al. (2019). This work is published and licensed by Dove Medical Press Limited and incorporate the Creative Commons Attribution—Non-Commercial (unported, v3.0) License (http://creativecommons.org/licenses/by-nc/3.0/).

Other enzyme-responsive MSN was modified with triphenylphosphine (TPP) for mitochondria targeting, DOX for anticancer activity and HA capping for CD44 targeting. The enzyme-responsive drug release was studied over time in the presence and absence of the HAase enzyme, which showed significant release of the drug from the nanoparticle when treated with the enzyme. The DOX-loaded final nanoparticle showed toxicity to stomach cancer MGC-803 cells. A live/dead assay was conducted to understand more about the cytotoxicity of the nanoparticles (Figure 4) (Naz et al., 2019).

### ROS-Responsive Systems

The generation and persistence of higher amounts of ROS was observed in cancer cells compared to normal cells due to the stimulation of different oncogenes, inflammation and mitochondrial damage (Li et al., 2020). A number of strategies have been developed for the design of ROS-responsive nanoparticle systems for cancer therapy by utilizing their physiochemical characteristics. The utilization of ROS-cleavable bonds in cargos for effective and sustained drug release in the presence of different hydroxyl radicals (^•^OH), hydrogen peroxides (H_2_O_2_), superoxide anions (${\text{O}}_{2}^{-}$), and peroxynitries (ONOO^−^) is an emerging field of research interest (Tao and He, 2018; Aggarwal et al., 2019). A number of studies have been reported that make use of different linkages for ROS-responsive drug release. The major types of the currently used linkers are thioethers, selenides, arylboronic esters, thioketals, aminoacrylates, oligoprolines, peroxalate esters, mesoporous silicons, etc. Drug release from the nanoparticles depends on the chemical structure and reaction mechanism of the linkers. The major parameters in the drug release mechanisms are ROS-induced dissolution of the carrier system, ROS-induced carrier breakage and prodrug breakage (Tao and He, 2018) (Table 4).

**Table 4:** ROS stimuli responsive nanoparticle for anti-cancer drug release.

**Nanoparticular components**	**Mechanism**	**Payload**	**References**
Arginylglycylaspartic acid	Degradation of thioketal group under the biological ROS level	EpothiloneB	Bertoni et al., 2020
Polyphatidylcholines	Degradation of thioether group under the biological ROS level	Doxorubicin	Yin et al., 2020
Poly([N-(2-hydroxypropyl)] methacrylamide	Degradation of boronic ester linker under the biological ROS level	Doxorubicin	Du et al., 2019
Polydimethylsiloxane	Mitochondrial ROS mediated destabilization	Camptothecin	Zhang W. et al., 2019
PEG grafted poly(acrylic acid)	Mitochondrial ROS mediated destabilization	Curcumin	Luo et al., 2017
PEG grafted poly glycolic acid	Degradation of thioketal group under the biological ROS level	Celastrol	Niu et al., 2020

Nanoparticles with thioketal (tk) linkers, which are sensitive to ROS stimuli, as well as the tumor-targeting peptide RGD with anticancer epothilone B (Epo B) showed an effective anticancer effect both *in vitro* and *in vivo*. Self-assembly of RGD-tk-EpoB resulted in a stable nanoparticle structure, which can undergo degradation via the tk group at the biological ROS level, which in turn leads to the release of the EpoB drug. An RGD targeted internalization and cell apoptosis study with PC-3 cells showed the efficacy of the nanoparticles to target the cells followed by induction of apoptosis (Xia et al., 2020). In another study, micelles were synthesized using the stimuli-responsive amphiphilic diblock copolymer prodrug, and the side chain contained poly(methacrylate) conjugated with the thioketal linker CPT. The final micellar particle GR-BCP exhibited a spherical morphology with ROS-responsive drug release when treated with biological amounts of H_2_O_2_ (Bertoni et al., 2020). The use of lipids as well as liposomes for drug delivery has been studied for different cancer treatments. The development of a tumor microenvironment ROS-responsive nanoparticle system using polyphatidylcholines S-PCs and liposomes loaded with doxorubicin DOX was studied. The thioether bond present in the final formulation is oxidized in the presence of ROS, which in turn results in drug release (Yin et al., 2020).

Another strategy for the release of drugs from the nanoparticles uses spacer chemistry to destabilize the nanoparticles in the TME. Novel ready-to-use amphiphilic block polymer-modified polymersomes (PS) were synthesized for the release of DOX. The tunable site-specific release of the drug was achieved through incorporation of a hydrophobic boronic ester-based ROS sensor into the backbone of the polymer. In addition, the reaction of ROS results in the production of hydrophilic carboxylic acid or phenol, which can destabilize DOX-loaded PS. Cumulative release of the drug also indicated the time-dependent sustained release of DOX (Du et al., 2019). Mitochondrial ROS can be used for the stimuli-responsive release of drugs for cancer therapy. Here, endogenous mitochondrial ROS was utilized to release the cellular respiration inhibitor CPT from dual targeting polydrug nanoreactors. This CPT can be used for the mitochondrial ROS burst. Cumulative drug release from the carrier nanoparticle was found to be increased after treatment with H_2_O_2_, indicating ROS-responsive drug release (Zhang W. et al., 2019). Celastrol is a water insoluble naturally derived anticancer drug found effective against ovarian cancer. For effective targeting and delivery of drug a thioketal ROS responsive linker based nanoparticle was developed with folic acid ovarian cancer targeting ability. Thioketal linker was added between PLGA and PEG polymer moiety. In final preparation a water in oil emulsion of celestrol containing nanoparticle containing PLGA-Tk-PEG and PVA was developed. Effective ROS stimulated breaking of thioletal linker was obtained and subsequent release of celastrol caused therapeutic effect in ovarian cancer (Niu et al., 2020).

**Figure 5:**
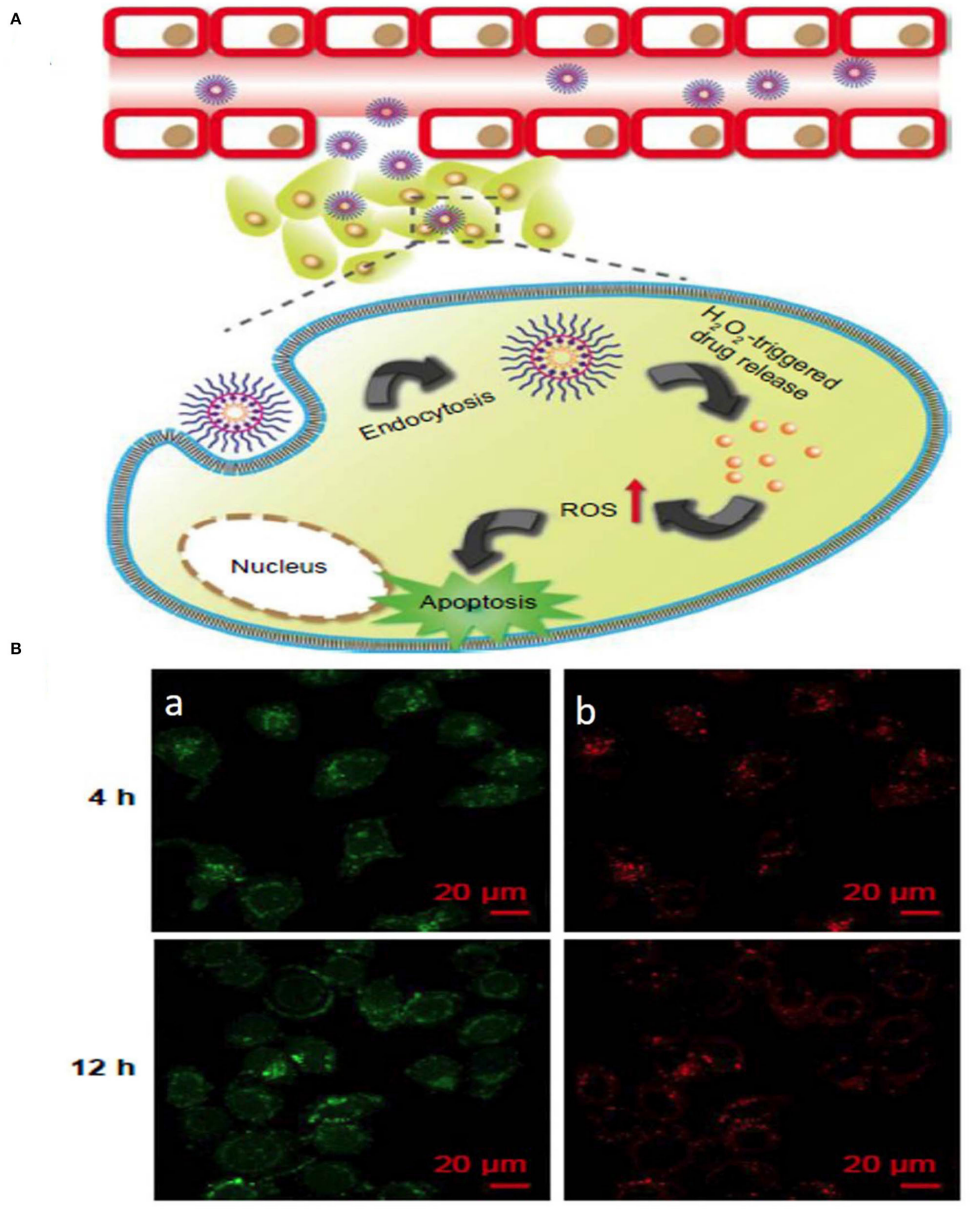
Schematic illustration of **(A)** ROS stimuli responsive delivery of PPHC nanoparticles, schematic representation of ROS triggered intracellular drug delivery followed by apoptosis. **(B)** Confocal microscopic images of intracellular localization of PPHC nanoparticles in A549 cells at 4 h and 12 incubation [(a) PPHC channel, (b) lysotracker red channel. Reprinted with permission from Luo et al. (2017). This work is published and licensed by Dove Medical Press Limited. The full terms of this license are available at https://www.dovepress.com/terms.php and incorporate the Creative Commons Attribution].

The interaction between curcumin and phenylboronic acid (PBA) can be used to fabricate ROS-responsive nanoparticles. Curcumin can be used to regulate growth inhibition and apoptosis in cancer cells. In a related study, a biocompatible PBA modified with PEG-grafted poly(acrylic acid) was used to synthesize TME ROS-responsive nanoparticles with encapsulated curcumin for effective delivery of curcumin to the target site. The H_2_O_2_-triggered curcumin release was investigated to understand the release pattern of the drug in the presence and absence of ROS (Figure 5) (Luo et al., 2017). Although ROS-responsive nanoparticle systems are a promising tool for delivering therapeutic cargos to the tumor site, the synergistic effect of ROS with temperature, enzymes, hypoxia, etc. could be used as an efficient method of treatment.

### Hypoxia-Responsive Systems

The TME plays a major role in causing hypoxia and in turn promoting cancer progression. Hypoxia is a phenomenon in tumors attributed to an insufficient amount of oxygen reaching the cells due to the blood supply disruption, usually in deeper parts of tumors. The hypoxic level in cells is defined as an oxygen threshold level of 2% or less from a normal oxygen level of 9% (Muz et al., 2015). Hypoxia is expressed in the majority of tumors and is also considered to be a prognostic factor associated with tumor progression (Jing et al., 2019). Utilizing hypoxia to enable nanoparticles to change their physiochemical properties to release drugs has been the subject of many studies. Similar to other stimuli release mechanisms, hypoxia-mediated release also depends on certain degradation mechanisms. The main mechanism requires reducible functional groups in the material, which can receive electrons and change the material physical properties, such as hydrophobicity, in the presence of hypoxia. Approximately three types of moieties are responsible for hypoxia-responsive release; azo linkers, nitrobenzyl alcohol, and nitroimidazoles. Nitroimidazole possesses bioreductive groups, which undergo reduction under hypoxic conditions and generate intermediates causing polymers to change their physiochemical properties, allowing the drug to be released. Nitrobenzyl alcohol induces electron transfer, and derivatives of nitrobenzyl are degraded by the 1–6 elimination reaction. Azobenzene also undergoes electron transfer and is reduced to aniline, which reduces the stability of the polymer structure and initiates drug release (Thambi et al., 2016) (Table 5).

**Table 5:** Hypoxia stimuli responsive nanoparticle for anti-cancer drug release.

**Nanoparticular components**	**Mechanism**	**Payload**	**References**
Polyethyleneglycol	Hypoxia sensitive p-nitrobenzyl derivative destabilization	Doxorubicin	Zhang et al., 2020
Poly-L-Lysine	Hypoxia sensitive nitrobenzyl chlorformate moiety destabilization	Doxorubicin	Thambi et al., 2016
Carboxymethyl dextran	Hypoxia sensitive azo bon destabilization	Doxorubicin	Son et al., 2018
Poly(N-isopropylacrylamide	Hypoxia sensitive azobenzene linker destabilization	Gemcitabine	Kulkarni et al., 2016
Human serum albumin	Hypoxia sensitive azobenzene linker destabilization	Oxaliplatin	Yang et al., 2019
Poly(lactic-co-glycolic acid)	Hypoxia sensitive azobenzene linker destabilization	Doxorubicin	Li Z. et al., 2018
Polyethyleneglycol, polylactic acid	Hypoxia sensitive azobenzene linker destabilization	Doxorubicin	Mamnoon et al., 2020

**Figure 6:**
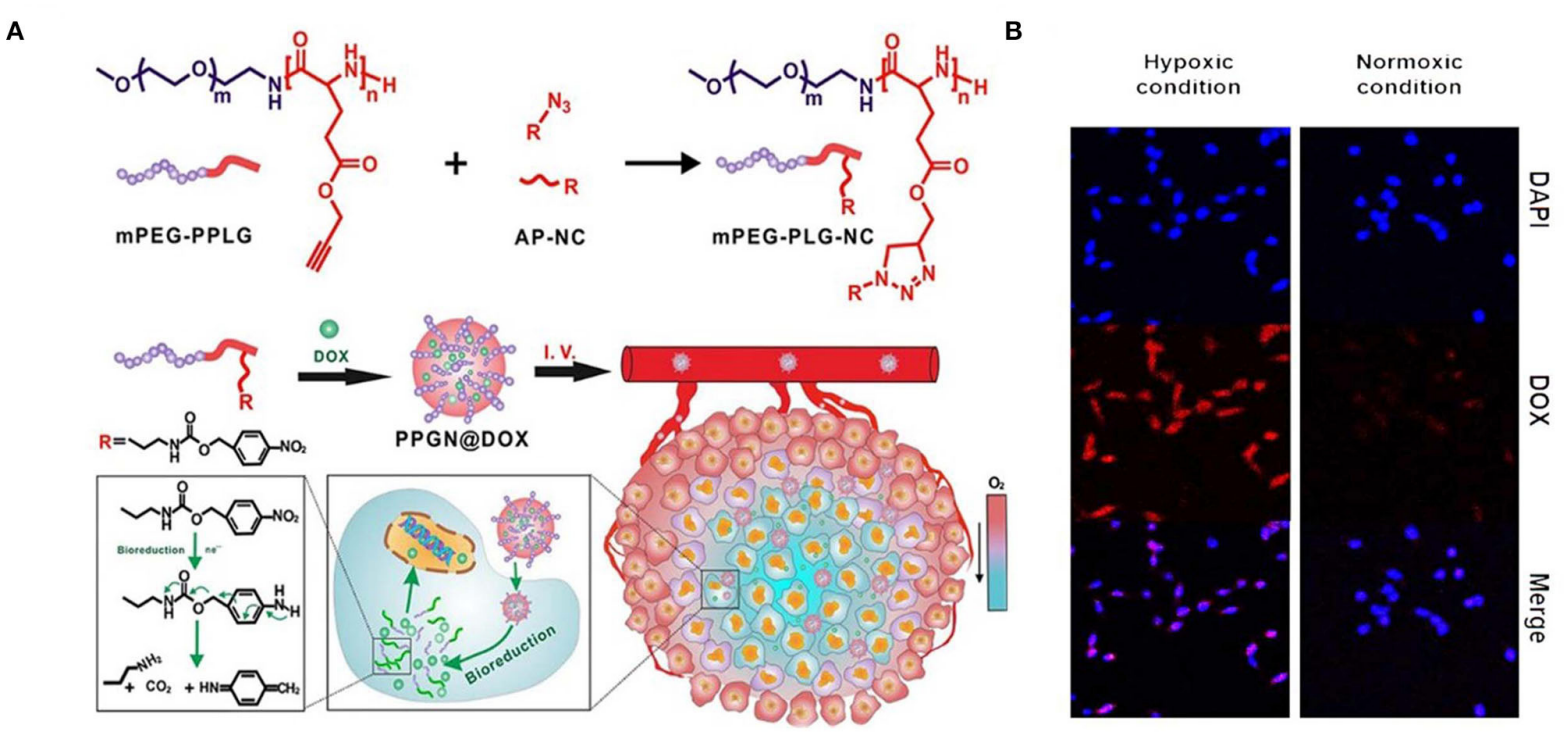
Schematic illustration of synthesis and intracellular hypoxia stimuli responsive delivery of PPGN@DOX nanoparticles. **(A)** Schematic representation of hypoxia triggered intracellular drug delivery. **(B)** Confocal microscopic images of intracellular localization of PPGN@DOX nanoparticles in cells at Hypoxa and Normoxia condition. Reprinted with permission from Zhang et al. (2020). Copyright © 2020 American Chemical Society.

A hypoxia-sensitive polymer based on a hydrophobic p-nitrobenzyl derivative, 4-nitrobenzyl (3-azidopropyl) carbamate (AP-NC) conjugated to methoxy PEG-b-poly(γ-propargyl-L-glutamate) (PPLG) copolymers self-assembled into micelles was developed to load DOX. The DOX release was enhanced in the hypoxic environment when the amino groups in the mPEG-PPLG-NC side chain were liberated after conversion of the p-aminobenzyl group. The p-nitrobenzyl derivative of AP-NC is sensitive to hypoxia and forms a reduced p-aminobenzyl group due to the overexpression of nitroreductase in a hypoxic environment (Figure 6) (Zhang et al., 2020). In a similar kind of application, an amphiphilic block copolymer, composed of PEG as the hydrophilic block and poly(ε-(4-nitro)benzyloxycarbonyl-L-lysine) as the hydrophobic block, was prepared for DOX. The DOX-loaded micelles exhibited rapid intracellular release of DOX under the hypoxic condition, implying high potential as a drug carrier for cancer therapy (Thambi et al., 2016). In another study, a hypoxia-sensitive azo-bond containing black hole quencher 3 was conjugated to carboxymethyl dextran to form nanoparticles with loaded DOX. *In vitro* cytotoxicity study revealed that this nanopartilces showed higher toxicity under hypoxic conditions than normoxic conditions (Son et al., 2018). Azobenzene-based nanoparticles linking PEG and PEI-DOPE (PAPD) were synthesized to load siRNA. In a hypoxic environment, the azobenzene linker is cleaved, and PEI-DOPE/siRNA is exposed and taken up by cells. Hypoxia-induced gene silencing was observed in the *in vitro* analysis (Perche et al., 2014). Human serum albumin (HSA)-based nanoparticles were prepared with a hypoxia-sensitive azobenzene linker connecting chlorin-e6 and oxaliplatin to HSA (Yang et al., 2019).

**Figure 7:**
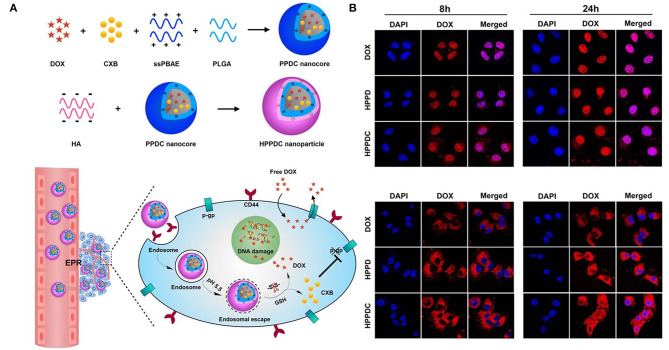
Schematic illustration of **(A)** synthesis and intracellular pH and ROS stimuli responsive delivery of HPPDC nanoparticles **(B)** Confocal microscopic images of intracellular localization of HPPDC nanoparticles in MCF7/ADR cells at (a) 8 h and (b) 24 h incubation. Reprinted with permission from Zhang S. et al. (2019). This work is published and licensed by Springer Nature under the terms of Creative Commons license.

The dissociated particles of HC (HSA+chlorin e6) and HO (HSA+oxaliplatin) enhanced the anticancer effect through photodynamic and chemotherapeutic mechanisms. The release of chorine 6 due to hypoxic-mediated release was measured indirectly by fluorescence, which showed a periodic increase in fluorescence in the nanoparticle formulation. This nanoparticle was tested in terms of cell uptake and response to hypoxic conditions, under which it showed enhanced release of doxorubicin compared with normoxic conditions. NP study showed the presence of DOX in the deep regions of spheroids/tumors due to hypoxic conditions enabling azobenzene linker disruption (Li Z. et al., 2018). Breast cancer have overexpressing 17β-Estradiol (E2) cells that provide unique opportunity for breast tumor targeting for drug delivery. Polymerosome loaded with doxorubicin was developed with estradiol targeting for hypoxic stimuli responsive breast cancer. Hypoxia environment (2% oxygen) stimulated release of almost 90% of drug release compared to 30% release of DOX (21% oxygen) (Mamnoon et al., 2020).

### Multistimuli-Responsive Systems

Multistimuli-responsive nanoparticle systems use a combination of internal tumor stimuli to release drugs from the nanoparticles (Figure 7). Compared to release in response to a single stimulus, drugs can be released from multistimuli nanoparticles in a programmed manner (Cheng et al., 2013) (Table 6). A triple stimuli-responsive system (pH/GSH/enzyme) was developed with keratin and DOX by an ionic gelation method. The release increased at low pH due to protonation of keratin and DOX. In a similar manner, GSH-based release was observed at GSH concentrations of 10 and 50 mM. Trypsin is overexpressed in the tumor microenvironment and therefore very useful as an enzyme stimulus for drug release. With the combined stimuli action of pH, GSH and trypsin, the cumulative release obtained was very high compared with that obtained through a single stimulus. Keratin DOX nanoparticles (KDNPs) have the potential to release nitric oxide (NO) in the presence of GSH. Overall, KDNPs are effective multistimuli platforms for the release of DOX and NO (Li Y. et al., 2018).

**Table 6:** Multi-stimuli responsive nanoparticle for anti-cancer drug release.

**Nanoparticular components**	**Mechanism**	**Payload**	**References**
Keratin	pH stimuli release by protonation of keratin/GSH/enzyme stimuli release by trypsin overexpression in TME	Doxorubicin	Li Y. et al., 2018
Poly(N-isopropylacrylamide	Hypoxia sensitive nitroimidazole (NI) group conversion to amino imidazole group/Temperature sensitive polymer PNIPAM	Doxorubicin	Ji et al., 2020
Poly(methacrylic acid), poly(N-isopropylacrylamide	pH sensitive PMAA/Temperature sensitive PNIPAM	Doxorubicin	Zheng et al., 2017
Poly(ethylene glycol)	Redox/pH sensitive selenocystine-acetyl histidine (Ac-histidine)	Piperlongumine	Lee et al., 2018
Mesoporous silica	Redox responsiveness from disulfide bond/pH sensitiveness from chitosan shell	Doxorubicin	Chen Y. et al., 2019
Poly(2- methacryloyloxyethyl phosphorylcholine, poly(L-histidine)	Redox sensitive p[MPC]/pH sensitive poly(L-histidine)	Doxorubicin	John et al., 2017
Poly(2-methacryloyloxyethyl phosphorylcholine, poly(L-aspartic acid	Redox responsiveness from disulfide bond/pH sensitive poly(β-amino ester)	Methotrexate	Zhang et al., 2018
Poly(2-methacryloyloxyethyl phosphorylcholine, poly(L-aspartic acid	Redox/pH responsive zwitterionic poly(2-methacryloyloxyethyl phosphorylcholine)50-block-poly(L-aspartic acid)n (p(MPC)50–b–p(AA)n)	Doxorubicin	Johnson et al., 2017
Poly(lactic-co-glycolic acid), poly(β-amino ester)	Redox responsiveness from disulfide bond/pH sensitive poly(β-amino ester)	Doxorubicin	Zhang S. et al., 2019

Random copolymers of (PNIPAM-co-AA-co-NIA) and poly(isopropylacryamide-co-acrylic acid-co-2-nitroimidazole acrylate) were self-assembled into spherical micelles at room temperature by EDC and RAFT reactions. The copolymers contained different levels of the 2-nitroimidazole (NI) group, which responds to hypoxic conditions by being transformed into an amino imidazole group (hydrophobic to hydrophilic transition), thereby releasing the drug. In addition, the temperature-sensitive property of PNIPAM can further facilitate the release of the drug. The cumulative release of DOX was found to be almost 55% in both hypoxic and high-temperature conditions and nearly 20% in normoxic and low-temperature conditions (Ji et al., 2020).

A pH- and thermal-based stimuli-responsive system was synthesized with PMAA and PNIPAM coated onto a silica core to form SiO_2_-PMAA-b-PNIPAM nanoparticles. The particle size was 15 nm, which was very small, enabling the incorporation of more drug and facilitating the deep penetration into the tissue. Apart from pH- and temperature-based release, the nanoparticles also possessed the ability to precipitate in acidic conditions at an increased temperature, which facilitated the accumulation of nanoparticles in the tumor tissue. *In vitro* studies were conducted with HeLa cells and revealed the uptake of the particles and drug release ability (Zheng et al., 2017). The stimuli-responsive behavior of the nanoparticles was utilized to treat lung metastasis of colorectal cancer cells using methoxy poly(ethylene glycol)-grafted chitosan crosslinked to selenocystine-acetyl histidine (Ac-histidine) conjugates (PL NPs). An *in vivo* study was performed in a pulmonary metastasis model using the CT-26 cell line. Lung metastasis in the PL NP-treated group was inhibited (Lee et al., 2018).

In another study, MSN-based redox/pH-responsive nanoparticles were developed with carbon dots, HA and chitosan as capping agents. This nanoparticle released the loaded DOX via a simple mechanism, which involved the destabilization of HA through the breakage of disulfide bonds to uncover chitosan and release the drug in response to the redox environment in the tumor (Chen Y. et al., 2019). pH/redox-responsive dox-loaded polymeric micelles were developed using poly(2- methacryloyloxyethyl phosphorylcholine) 25-block-poly(L-histidine) n (p[MPC])25-b-p[His]n, with p[His] as the pH-responsive element and p[MPC] as a redox-responsive block (John et al., 2017). A charge reversible pullulan derivative was used to develop a pH- and redox-responsive nanoparticle. Methotrexate, a cancer drug, was conjugated to an amino-modified poly(β-amino ester) amino ester. The nanoparticles could also load plasmid DNA coding for cancer therapeutic effects (Zhang et al., 2018). A pH-responsive zwitterionic poly(2-methacryloyloxyethyl phosphorylcholine)50-block-poly(L-aspartic acid)n (p(MPC)50–b–p(AA)n) was synthesized with a disulfide linker for dual stimuli-induced drug release of DOX. DOX release also seemed to be very high, when incubated in 4T1 cells at pH 5.5 (Johnson et al., 2017). Hyaluronic acid based nanoparticles were synthesized for loading multi drug resistance (MDR) blocking drug (Cyclooxygenase 2) and doxorubicin to treat against MDR resistant tumor. This nanoparticle contain core of sPBAE, PLGA Cyclooxygenase 2 (CXB) and doxorubicin (Zhang S. et al., 2019).

Internal stimuli in combination with external stimuli, such as light, temperature, or mechanical stimuli can enhance the release of the drug. In most cases, the TME itself may provide one kind of stimulus to facilitate drug release, but this approach might not be sufficient for the drug release from certain kinds of nanoparticles. Therefore, external stimuli can provide an additional push to reach the maximum release potential of the drug. However, a disadvantage of external stimuli-responsive materials is their inability to reach the site of the tumor to induce the appropriate release of the drug. For instance, photodynamic or photothermal stimuli can diffuse inside the body before reaching the active site. Regarding future use, there is a need to combine polymers, inorganic materials, and lipids to design multistimuli responsive anticancer drug delivery systems (An et al., 2016).

## Summary and Future Directions

The main characteristic of stimuli-responsive drug delivery systems compared to the direct delivery of small-molecule anticancer drugs is their ability to release the drug in a stimuli-responsive manner, high drug loading capability and biocompatibility. The internal stimuli are activated inside the tumor. An internal stimuli-responsive system depends on the variation of stimuli, such as enzymatic variations, GSH level, pH, and ROS in the TME. This capability of internal stimuli release systems needs further development to gain more value than external stimuli responsive systems. In the field of nanotechnology, the new findings have paved a way for exclusive designs of polymeric nanosystems along with the capability for tuning their chemistry based on the surface and characterization of their physical nature, such as shape and size, to induce stimuli-responsiveness. Clinical translation of external stimuli responsive nanoparticles is conducted worldwide. But there are only limited clinical trial studies on internal stimuli responsive nanoparticle system such as pH responsive polymeric micelle loaded with epirubicin (NCT03168061) which is undergoing phase 1 and phase 2 trials. Another one is cisplatin loaded liposomal system (NCT01861496) which is internally activated by secretory phospholipase undergoing phase 1 and phase 2 trials (Mi, 2020).

An internal stimuli-responsive system should be developed which can respond to biochemical signals in the TME at very low concentrations in the range of nanomoles. This system would ensure a higher drug release efficacy of the nanoparticle system. This system would also open the possibility for incorporating a programmed multistage drug release mechanism involving biological processes. Overall, the future of internal stimuli-responsive systems depends on programmable and smart nanoparticle carriers, which can load and release drugs at the target site with maximum efficiency (An et al., 2016).

## Author Contributions

RT and SS wrote the manuscript. YJ wrote and edited the manuscript. All authors contributed to the article and approved the submitted version.

## Conflict of Interest

The authors declare that the research was conducted in the absence of any commercial or financial relationships that could be construed as a potential conflict of interest.
