# Corrigendum: Tumor Microenvironment-Stimuli Responsive Nanoparticles for Anticancer Therapy

**DOI:** 10.3389/fmolb.2021.693909

**Published:** 2021-05-06

**Authors:** Reju George Thomas, Suchithra Poilil Surendran, Yong Yeon Jeong

**Affiliations:** ^1^Department of Radiology, Chonnam National University Hwasun Hospital, Hwasun, South Korea; ^2^BioMolecular Theranostics (BiT) Laboratory, Department of Biomedical Sciences, Chonnam National University Medical School, Chonnam National University Hwasun Hospital, Gwangju, South Korea

**Keywords:** stimuli, nanoparticle, tumor microenvironment, cancer, drug release

There is an error in the Funding statement. The correct number and name are as follows:

This work was supported by the Technology Innovation Program (or Industrial Strategic Technology Development Program-Development of Core Industrial Technology) (20003822, Development of Navigation System Technologies of MicroNano Robots with Drug for Brain Disease Therapy) funded by the Ministry of Trade, Industry & Energy (MOTIE, Korea).

The authors apologize for this error and state that this does not change the scientific conclusions of the article in any way. The original article has been updated.

